# PostOperative ST‐segment elevation: not a blocked coronary artery, then what?

**DOI:** 10.1002/ccr3.1116

**Published:** 2018-02-16

**Authors:** Wai‐Ching Sin, Joy Melody Kwong, Tiffany Cho‐Lam Wong, Charlotte Kwong, Carmen Chan, Chung‐Wah Siu

**Affiliations:** ^1^ Department of Adult Intensive Care Queen Mary Hospital Hong Kong China; ^2^ Department of Surgery Li Ka Shing Faculty of Medicine The University of Hong Kong Hong Kong China; ^3^ Department of Diagnostic Radiology Queen Mary Hospital Hong Kong China; ^4^ Division of Cardiology Department of Medicine Li Ka Shing Faculty of Medicine The University of Hong Kong Hong Kong China

**Keywords:** Computer tomography coronary angiography, myocardial infarct, pneumomediastinum, ST‐segment elevation

## Abstract

ST‐segment elevation is well known for its diagnostic value for transmural myocardial infarction due to acute thrombotic occlusion of a coronary artery, and often requires emergency reperfusion therapy. However, ST segment is by no means pathognomonic for acute coronary events. Here, we report a case of ST‐segment elevation after hepatectomy secondary to an unusual etiology.

## Case History

**Figure 1:**
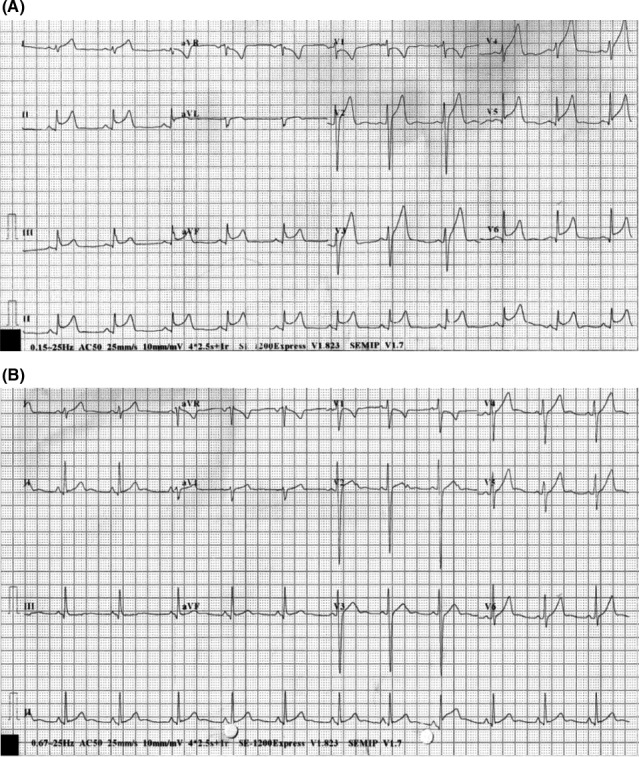
Twelve‐lead electrocardiogram on day 1 (A) and day 5 (B).

**Figure 2:**
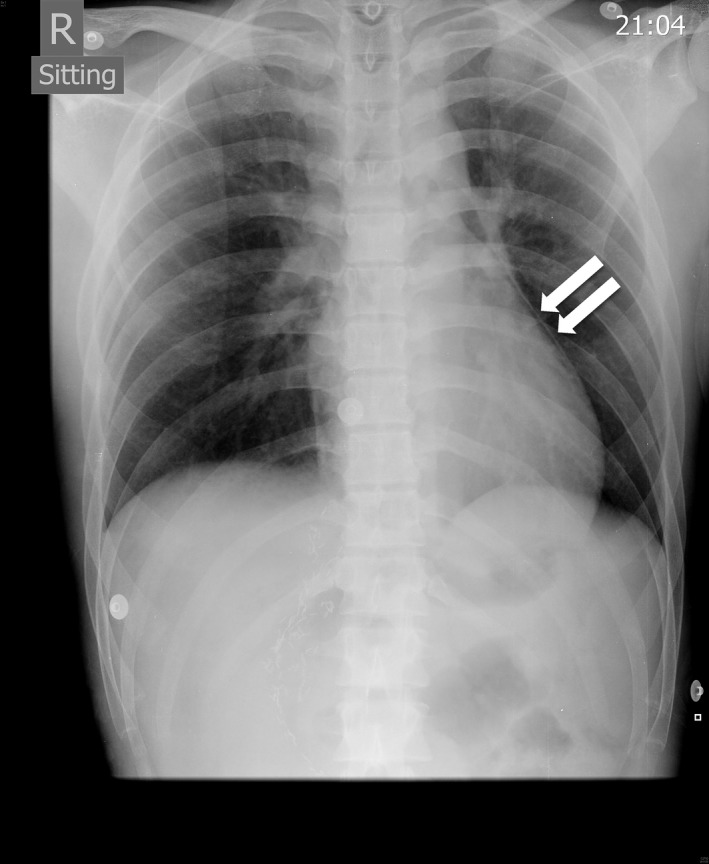
Chest radiograph (arrows indicate a radiolucency behind the heart).

**Figure 3:**
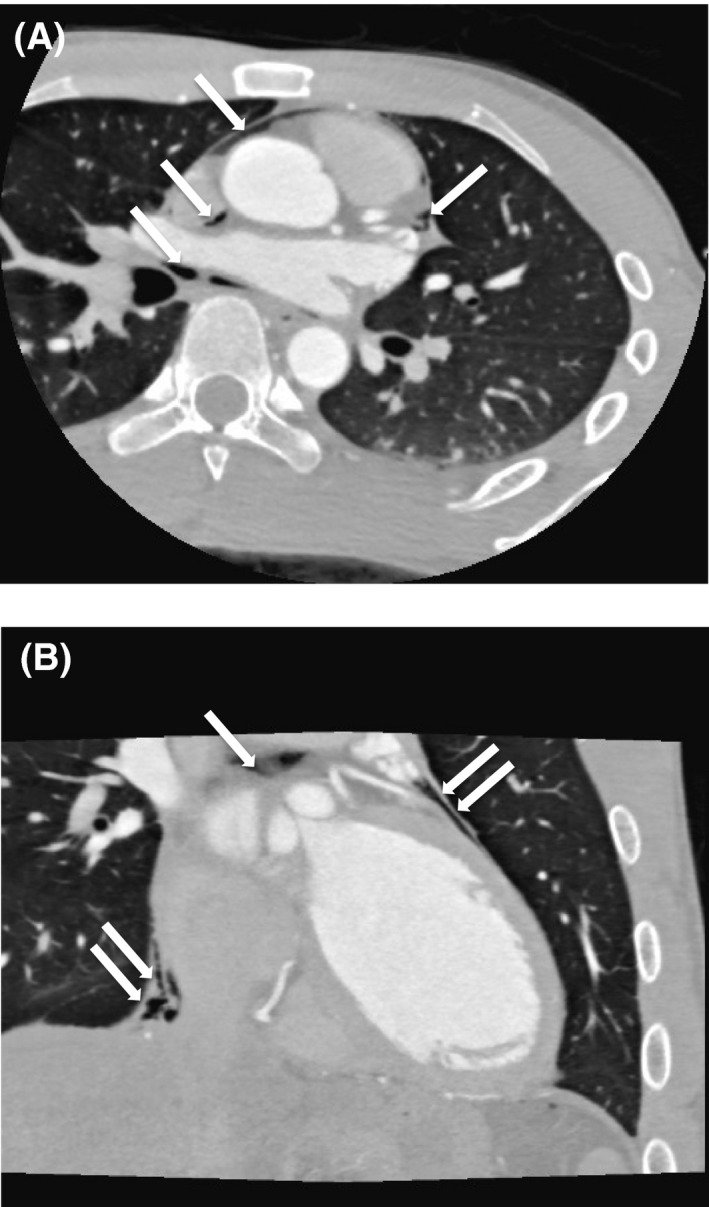
Computed tomography of the thorax: (A) coronal view and (B) sagittal view (arrows indicate air within mediastinum and pericardial space).

A 22‐year‐old healthy man was transferred postoperatively to our intensive care unit following elective hepatectomy of the left and caudate lobe for live‐donor, liver transplantation. The procedure was uneventful. He was a nonsmoker and nondrinker. He had no significant personal medical history, or family history of premature cardiovascular disease or sudden cardiac death. On arrival, he had been extubated and blood pressure was 98/62 mmHg and pulse rate 86 bpm. No inotropic support was required. Physical examination did not reveal any abnormality suggestive of rheumatological conditions or familial hypercholesterolemia. Routine electrocardiogram (ECG) 8 h later revealed sinus rhythm at a rate of 65 bpm. Nonetheless, there were new widespread ST‐segment elevations over the inferior leads (II, III, aVF) and anterolateral precordial leads (V2–V6; Fig. 1). PR‐segment depression was also noted over leads II, III and aVF. He was asymptomatic and hemodynamically stable. Echocardiogram showed normal systolic function with a left ventricular ejection fraction of 65%. There was no pericardial effusion or any regional wall motion abnormality. Liver and kidney function tests together with serum electrolytes were all normal. However, an initial troponin I level of 0.03 ng/mL (normal range: <0.04 ng/mL) subsequently rose to 1.59 ng/mL. Chest radiograph in the anteroposterior projection (Fig. 2) revealed a radiolucent line at the left heart border. Computed tomography coronary angiogram was performed but showed no evidence of coronary artery disease or pericardial effusion. Nonetheless, the presence of air was confirmed in the mediastinal as well as the pericardial space (Fig. 3). The patient remained asymptomatic and hemodynamically stable; he was treated conservatively. The ST‐segment elevations resolved completely 5 days later (Fig. 1). Repeated chest radiograph on day 7 showed no residual pneumomediastinum or pneumopericardium. The patient was discharged 8 days after the operation.

## Discussion

ST‐segment elevation is well known for its diagnostic value for transmural myocardial infarction due to acute thrombotic occlusion of a coronary artery, and often requires emergency reperfusion therapy. Nonetheless, ST‐segment elevation is by no means pathognomonic for ST‐segment elevation myocardial infarction (STEMI); nonischemic ST‐segment elevation (NISTE) may be observed in a number of clinical conditions such as pericarditis (Table 1). In our case, the diffuse ST‐segment elevations that involved multiple coronary territories, the normal left ventricular ejection fraction on echocardiograph, together with the disproportionally low troponin level favored an alternative diagnosis to STEMI. The clinical presentation was consistent with that of pericarditis, but the short time period since surgery and the lack of pericardial effusion on echocardiogram also made pericarditis an unlikely diagnosis. Albeit rare, pneumomediastinum and pneumopericardium have occasionally been reported due to blunt chest trauma, barotrauma from mechanical ventilation, laparoscopic procedures, or other surgical procedures that breech the integrity of the diaphragm 1, 2, 3, 4. Review of our patient's operative record revealed that he had been ventilated at a low pressure throughout the operation, and the diaphragm had not been manipulated. The exact cause of the pneumomediastinum and pneumopericardium could not be determined in our patient. Nonetheless, this case demonstrates that these two uncommon conditions may underlie postoperative ST‐segment elevation.

## Authorship

W‐CS: drafted the manuscript and conceived the case. JMK: drafted the manuscript. TC‐LW: drafted the manuscript. CK: drafted the manuscript and interpreted the image. CC: interpreted the image. C‐WS: revised the manuscript and conceived the case.

**Table 1:** Causes of ST‐segment elevation

	Causes
Myocardium	ST‐segment elevation myocardial infarction
	Takotsubo cardiomyopathy
	Left ventricular hypertrophy
	Chronic left ventricular aneurysm
Conductive system	Left bundle branch block
	Early repolarization
Pericardium	Acute pericarditis
	Pneumopericardium
Systemic causes	Electrolyte disturbances: hyperkalemia and hypercalcemia

## Conflict of Interest

No author has a real or perceived conflict of interest or has received any personal or financial support.
